# Fasudil attenuates soluble fms-like tyrosine kinase-1 (sFlt-1)-induced hypertension in pregnant mice through RhoA/ROCK pathway

**DOI:** 10.18632/oncotarget.22017

**Published:** 2017-10-24

**Authors:** Ying Gu, Yaling Feng, Jinjin Yu, Hua Yuan, Yongxiang Yin, Jian Ding, Jun Zhao, Yaohui Xu, Jianjuan Xu, Haisha Che

**Affiliations:** ^1^ Department of Obstetrics and Gynecology, Wuxi Maternal and Child Health Hospital Affiliated to Nanjing Medical University, Wuxi, Jiangsu 214002, PR China; ^2^ Department of Obstetrics and Gynecology, The Affiliated Hospital of Jiangnan University (Wuxi Fourth People’s Hospital), Wuxi, Jiangsu 214062, PR China; ^3^ Department of Pathology, The Affiliated Maternity and Child Health Hospital of Nanjing Medical University, Wuxi, Jiangsu 214002, PR China

**Keywords:** preeclampsia, fasudil, endothelial dysfunction, hypertension, RhoA/ROCK

## Abstract

Preeclampsia (PE) has become the leading cause of maternal and fetal morbidity and mortality in the world, which is characterized by a systemic maternal inflammatory response associated with endothelial dysfunction, hypertension, and proteinuria. The development of PE is still barely predictable and thus challenging to prevent and manage clinically. Fasudil (FSD), the first-generation Rho/ROCK inhibitor, has been studied widely and applied in clinical practice with high safety and efficacy in treating hypertension and other cardiovascular diseases. However, few studies have focused on the effect of fasudil on preeclampsia *in vivo* and *in vitro*. Therefore, the aim of this study is to investigate the effects of fasudil on hypoxia/reoxygenation injury *in vitro* and its role on preeclamptic animal model. Here, we found that RhoA/ROCK pathway was significantly activated in H/R-challenged endothelial cells and in placenta and umbilical vessel of PE mice. And fasudil pre-treatment can protect vascular endothelial cells from H/R-induced apoptosis. In addition, inhibition of RhoA/ROCK pathway with fasudil can reduce the high blood pressure and urine protein levels as well as the concentration of s-Flt in peripheral and umbilical blood in a dose-dependent manner, thus resulting in prevention of the development of PE. Thus, Fasudil attenuates soluble fms-like tyrosine kinase-1 (sFlt-1)-induced hypertension in pregnant mice through RhoA/ROCK pathway, which would become a potential strategy for PE therapy.

## INTRODUCTION

Preeclampsia has become the leading cause of maternal and fetal morbidity and mortality in the world accounting for nearly 40% of births delivered at early gestation, which is characterized by a systemic maternal inflammatory response associated with endothelial dysfunction, hypertension, and proteinuria [1]. There is also evidence that the risk of subsequent cardiovascular disease is significantly increased in women affected by preeclamptic pregnancies [2]. Although the development of PE is still barely predictable and thus challenging to prevent and manage clinically, there is evidence that placental oxidative stress attributable to abnormal uteroplacental blood circulation plays a critical role [3].

Oxidative stress of the placenta is able to result in endothelial dysfunction. Several important antioxidants, such as superoxide dismutase (SOD) and glutathione peroxidase (GPx), are significantly decreased in the maternal circulation of women with preeclampsia [4]. A simple *in vitro* model wherein endothelial cell monolayers are exposed to hypoxia/reoxygenation (H/R) is widely used to mimic the microvascular dysfunction that is induced by oxidative stress [5]. And administration of antioxidant vitamins C and E can block H/R-mediated sFlt-1 secretion involving in inhibition of p38 signaling, and increase the secretions of sFlt-1 have been shown to participate in the pathogenesis of pre-eclampsia [6, 7]. Our previous study has demonstrated that exogenous alpha-1 antitrypsin alleviated hypoxia/reoxygenation injury in a dose- and time- dependent manner via inactivation of Rac1/p38 signaling and suppression of oxidative stress [8]. And exogenous alpha-1 antitrypsin injection increases the antioxidants and suppresses oxidative stress, and subsequent prevention of PE development [9]. Thus, antioxidant treatment may be a potential strategy for preeclampsia therapy.

It was reported that H/R-induced changes in endothelial permeability result from coordinated actions of the Rho GTPases Rac1 and RhoA, suggesting that Rho GTPases act as key mediators coupling cellular redox state to endothelial function [10]. Ras homolog gene family, member A (RhoA) was expressed in syncytiotrophoblasts and cytotrophoblasts. The results of immunochemistry staining and qPCR showed that RhoA protein and mRNA expression in placental tissues of mild and severe preeclampsia groups was significantly higher than that in normal pregnancy, indicating that increased expression of RhoA in placental tissues might play an important role in the pathogenesis of preeclampsia [11]. During preeclampsia, the release of reactive oxygen species might activate the RhoA kinase pathway to enhance vascular reactivity. Pretreatment with superoxide dismutase/catalase to quench reactive oxygen species or RhoA kinase inhibitor blocked enhanced responses in preeclamptic and normal vessels [12]. Fasudil (FSD), the first-generation Rho/ Rho-associated protein kinase (ROCK) inhibitor, has been studied widely and applied in clinical practice with high safety and efficacy in treating hypertension and other cardiovascular diseases [13]. Butruille L et al showed that fasudil exposure during late gestation alleviates the growth of intrauterine growth-restricted fetuses from hypertensive rat mothers [14]. However, few studies have focused on the effect of fasudil on preeclampsia *in vivo* and *in vitro*. Therefore, the aim of this study is to investigate the effect of fasudil on hypoxia/reoxygenation injury *in vitro* and its role on preeclamptic animal model.

## RESULTS

### Fasudil protects HUVEC cells from hypoxia/reoxygenation induced apoptosis

**Figure 1:**
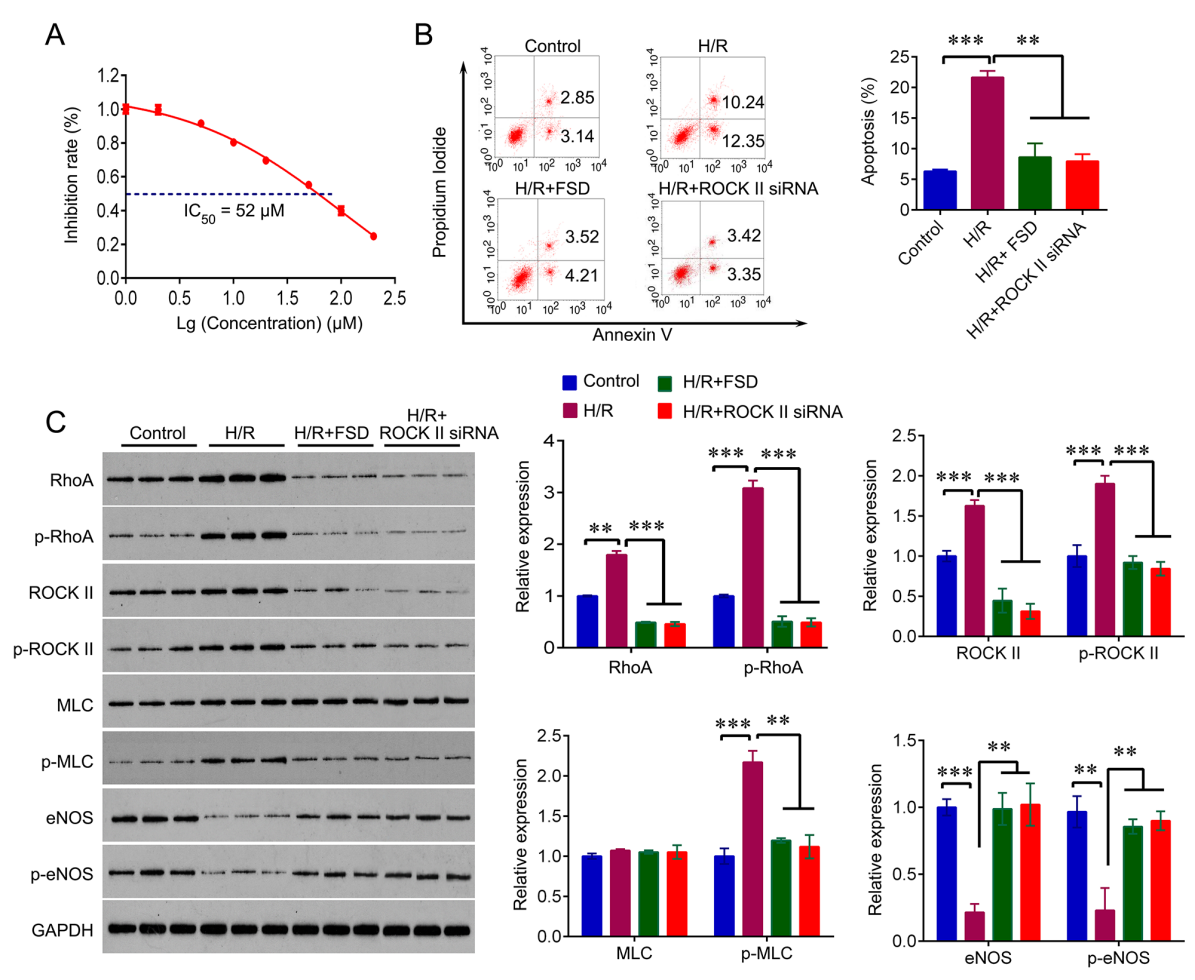
Fasudil protects HUVEC cells from H/R-induced apoptosis **(A)** MTT was used to determine the IC_50_ of FSD in HUVEC cells cultured in standard condition. **(B)** Flow cytometry was used to measure the apoptosis rate in HUVEC cells with indicated treatment. HUVEC cells cultured in standard condition were used as control. **(C)** Western blot analysis for RhoA, ROCK, MLC, eNOS and their phosphorylated form in HUVEC cells with indicated treatment. The experiments were independently repeated for three times. The experiments were independently repeated three times. Data were expressed as mean ± standard error. ANOVA with post hoc Tukey’s test was used for statistical analyses. H/R, Hypoxia-reoxygenation; FSD, fasudil. ^*^p<0.05, ^**^p<0.01, ^***^p<0.001.

We firstly determined the IC_50_ of Fasudil (FSD) in HUVEC cells. HUVEC cells were treated with a range of concentrations of Fasudil for 8 hr, and MTT were used to measure the cell viability. As shown in Figure 1A, the cell viability of HUVEC was decreased with increasing of concentrations of Fasudil; and the IC_50_ was determined at 52 μM.

We then used a well-established model of hypoxia/reoxygenation (H/R) treatment that causes vascular endothelial cells injury in HUVEC cells to investigate the role of Fasudil in H/R injury. HUVEC cells underwent 4 hr of hypoxia followed by 18 hr of reoxygenation. We pre-treated HUVEC cells with 50 μM Fasudil for 8 hr before H/R challenge. And the cells with no treatment were used as control. Exposure of HUVEC cells to H/R led to a significant increase in cell apoptosis (22.5%), while Fasudil pre-treatment significantly decreased percentage of apoptosis (7.7%) undergoing H/R challenge (Figure 1B), suggesting a protective effect of Fasudil against H/R injury. We further confirmed that inhibition of ROCK II by using siRNA markedly reduced the apoptosis induced by H/R (Figure 1B). In addition, we also analyzed the expression of key molecules of RhoA signaling, including RhoA, ROCK II, MLC and eNOS. We found that H/R treatment significantly upregulated RhoA and ROCK II expression and their phosphorylated form, and increased the expression of phosphorylated MLC, whereas reduced the expression of eNOS and p-eNOS. However, Fasudil pre-treatment and ROCK II knockdown reversed H/R-mediated effects on these molecules (Figure 1C), indicating that protective effect of Fasudil against H/R injury is involved in RhoA signaling.

### Fasudil attenuates the hypertension induced by sFlt-1 in pregnant mice

**Figure 2:**
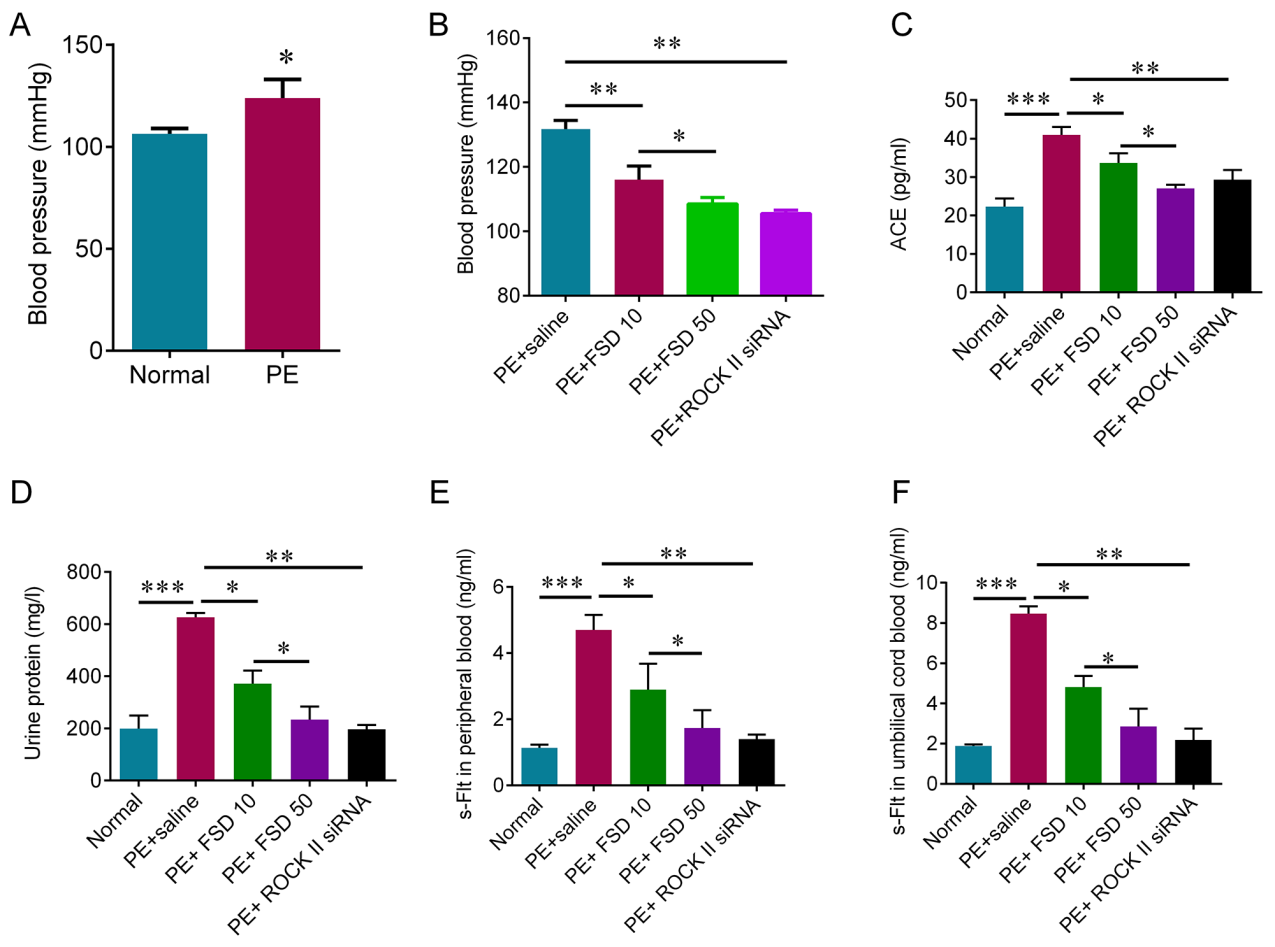
Fasudil decreases blood pressure induced by sFlt infusion in pregnant mice **(A)** Non-invasive rat tail blood pressure detecting method was used to measure blood pressure on day 13 of gestation. Student t test was used for statistical analyses. **(B)** Non-invasive rat tail blood pressure detecting method was used to measure systolic blood pressure on day 19 of gestation after 6 days Fasudil treatment or ROCK knockdown by siRNA. **(C)** ELISA was used to detect the concentration of Angiotensin converting enzyme (ACE) in peripheral blood. **(D)** The proteinuria was measured on day 19 of gestation after 6 days Fasudil treatment or ROCK knockdown by siRNA. The PE mice treated with saline were used as control. ELISA was used to detect the concentration of s-Flt in peripheral blood **(E)** and cord blood **(F)**. Number of animal for each group=8. Data were expressed as mean ± standard error. ANOVA with post hoc Tukey’s test was used for statistical analyses. ^*^p<0.05, ^**^p<0.01, ^***^p<0.001.

The results showed that sFlt-1 infusion significantly increased blood pressure on day 13 of gestation (Figure 2A), and elevated the urine protein levels compared with the normal group (Figure 2D), indicating that the PE model was successfully established. To investigate the role of Fasudil on PE, we injected Fasudil solution (10 or 50 mg/kg) into tail veins every day from days 13 to 19 of gestation in PE mice, and 100 μl of saline injection was used as a control. We found that Fasudil injection significantly relieved the high blood pressure, and reduced urine protein levels in a dose-dependent manner (Figure 2B and 2D). In addition, we measured the circulating ACE expression using ELISA kit, and found that ACE level was significantly increased in PE mice compared with normal mice, and inhibiting ROCK with fasudil or with ROCK II siRNA can reduce circulating ACE expression (Figure 2C). We also observed the fact that the concentrations of s-Flt in serum from both peripheral blood and umbilical cord blood were significantly increased in PE mice (Figure 2E and 2F). As expected, the concentrations of s-Flt in serum from both peripheral blood and umbilical cord blood were reduced with increased dosage of Fasudil (Figure 2E and 2F). In addition, knockdown of ROCK II by siRNA significantly relieved the high blood pressure, and reduced urine protein levels as well as the concentrations of s-Flt in serum from both peripheral blood and umbilical cord blood (Figure 2). Thus, these results suggest that Fasudil injection is able to relieve the symptoms of PE mediated by sFlt infusion, which may be through inactivating ROCK pathway.

### Fasudil inactivates RhoA signaling that induced by PE

**Figure 3:**
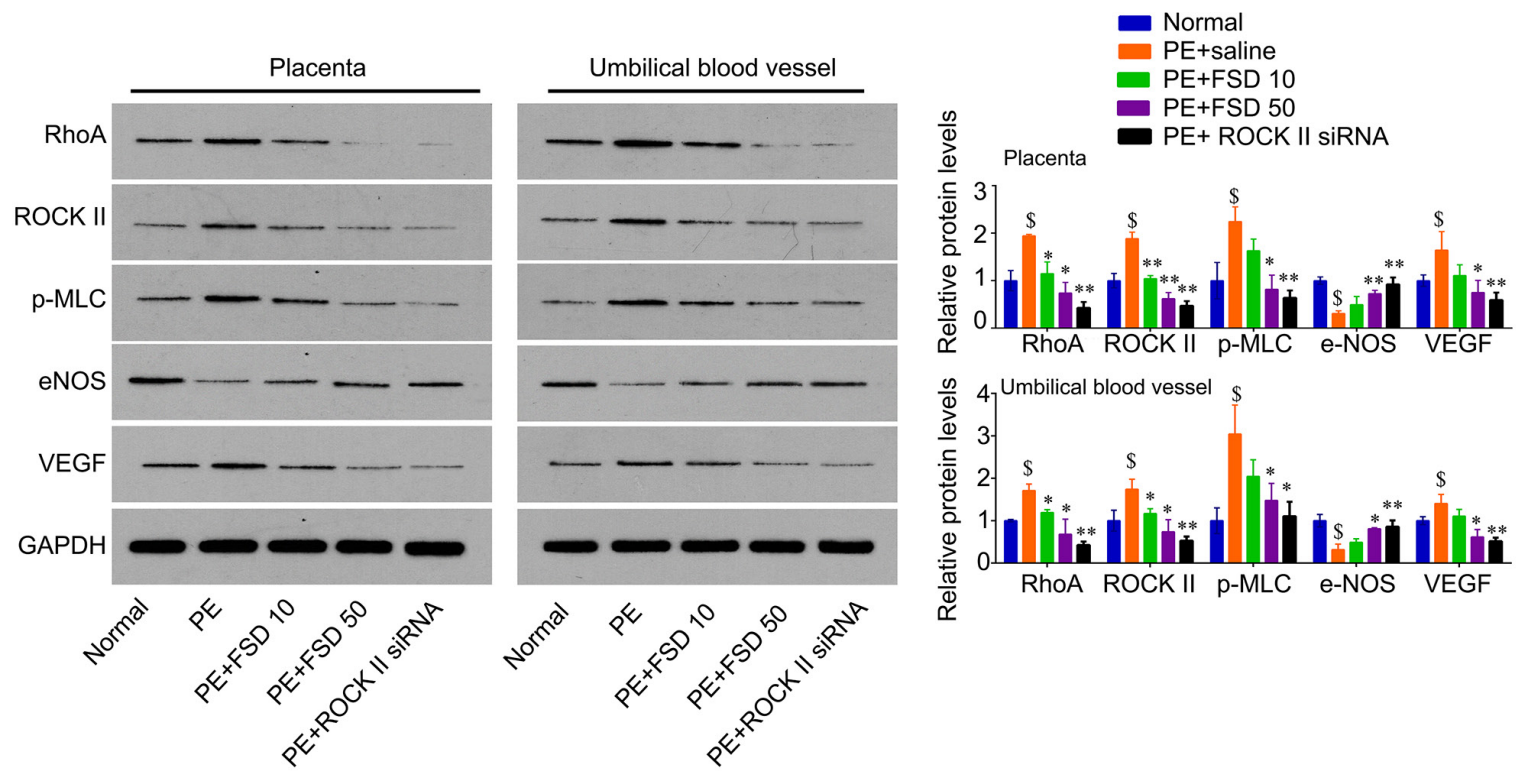
Fasudil regulates the expression of RhoA, ROCK, p-MLC, eNOS and VEGF in placenta and umbilical vessel from PE mice Western blot analysis for RhoA, ROCK, p-MLC, eNOS and VEGF in mice placenta and umbilical vessel, and quantification. Number of animal for each group=8. Data were expressed as mean ± standard error. ANOVA with post hoc Tukey’s test was used for statistical analyses. $p<0.05 *vs*. Normal. ^*^p<0.05, ^**^p<0.01 *vs.* PE+saline group.

**Figure 4:**
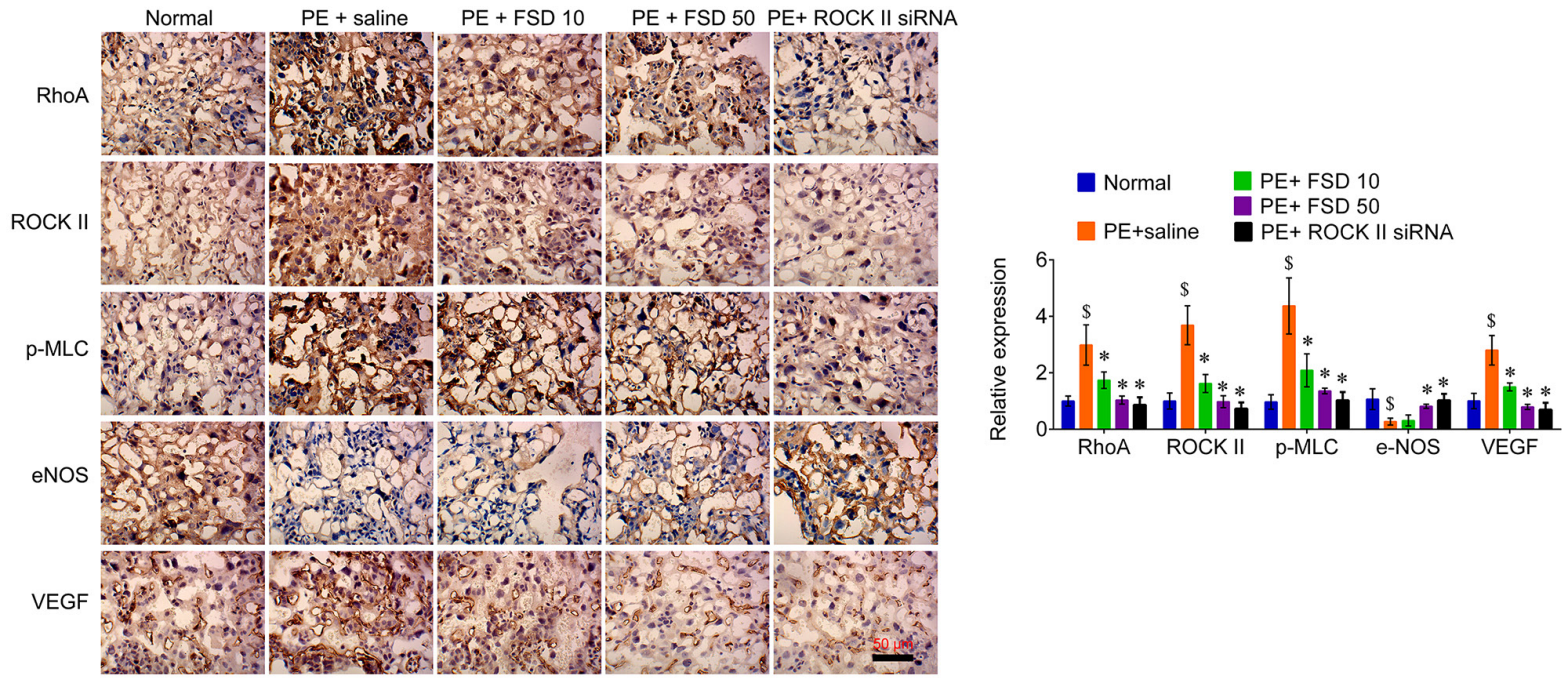
IHC analyzes the expression of RhoA, ROCK, p-MLC, eNOS and VEGF in placenta from PE mice Representative images of IHC staining for RhoA, ROCK, p-MLC, eNOS and VEGF (left), and quantification (right). Bar=50 μm; magnification, 200×. Number of animal for each group=8. Data were expressed as mean ± standard error. ANOVA with post hoc Tukey’s test was used for statistical analyses. $p<0.05 *vs*. Normal. ^*^p<0.05, ^**^p<0.01 *vs.* PE+saline group.

In addition, we obtained the placenta tissues from the pregnant mice to analyze Fasudil related downstream molecules. We found that PE activated the RhoA/ROCK signaling that enhanced the levels of these genes, including RhoA, ROCK II, p-MLC, as well as VEGF, but attenuated the levels of eNOS in both placenta and umbilical vessel tissues. We demonstrated that Fasudil was able to reduce the levels of RhoA, ROCK II, as well as VEGF, and the phosphorylation levels of MLC, but upregulated the levels of eNOS (Figure 3). Furthermore, we performed IHC assay to confirm that Fasudil injection significantly increased the expression of eNOS, and decreased the levels of RhoA, ROCK II and VEGF, as well as the phosphorylation levels of MLC that upregulated by PE in placenta tissues (Figure 3). And knockdown of ROCK II exerted the similar effects with Fasudil (Figures 3 and 4), indicating that prevention of the development of preeclampsia by Fasudil is involved in RhoA signaling.

## DISCUSSION

In this study, we at first time demonstrate that fasudil pre-treatment can protect vascular endothelial cells from H/R-induced apoptosis, and fasudil injection is able to relieve the high blood pressure and reduce urine protein levels in a dose-dependent manner, and thus prevent the development of PE.

H/R, similar to ischemia-reperfusion, triggers cells damage due to augmented oxidative stress, mitochondrial dysfunction and inflammation [15]. A deleterious role of activated RhoA/ROCK signaling in ischemia-reperfusion injury has been demonstrated in several *in vivo* models [16-18]. ROCK inhibition with fasudil achieved less inflammation, reduced oxidative stress and lower apoptosis, which may be involved in activation of the PI3K/Akt/eNOS signaling pathway, preservation of endothelial function and suppression of inflammatory responses [13, 19, 20]. In line with previous studies, we also found that fasudil pre-treatment can decrease H/R-induced apoptosis in vascular endothelial cells, which may through inhibition of RhoA/ROCK signaling and activation of eNOS signaling pathway [21].

Hypertension and oxidative stress are two hallmarkers of preeclampsia. Increased activity of RhoA/ROCK pathway has been observed in experimental hypertension models and hypertensive patients, which appear to be the consequence of the up-regulation of renin-angiotensin-aldosterone system and the increased production of reactive oxygen species (ROS) [22, 23]. ROCK, including two members ROCK1 and ROCK2, is one of the best-characterized effectors of small GTPase RhoA, which is involved in a wide range of fundamental cellular functions such as contraction, adhesion, migration, proliferation, and apoptosis [24, 25]. And ROCK is best known for promoting actin filament stabilization and generation of actin-myosin contractility by phosphorylating numerous downstream target proteins, including the myosin binding subunit of myosin light chain (MLC) [26-28]. Emerging evidence has demonstrated the potential therapeutic application of ROCK inhibitors in cardiovascular diseases with a remarkable efficacy in reducing vascular smooth muscle cell hypercontraction, endothelial dysfunction, inflammatory cell recruitment and vascular remodeling [29].

Genetic studies also support the importance of the RhoA/ROCK pathway in blood pressure [30, 31]. ROCK inhibition by fasudil reduces smooth muscle contractility through decreasing MLC phosphorylation and improving endothelial function via restoring eNOS expression/activity and NO production [21]. ROCK inactivation also reduces inflammation and remodeling through suppressing the expression of pro-inflammatory cytokines in endothelial and inhibiting reactive oxygen species (ROS) production [32, 33]. Increased blood pressure in preeclampsia is associated with marked vascular inflammation and ROS that may enhance vascular reactivity via the activation of RhoA/ROCK pathway [12]. Fasudil exposure during late gestation alleviates the growth of intrauterine growth restriction (IUGR) fetuses from hypertensive rat mothers, which may result from an improvement of maternal blood pressure induced by Fasudil [14]. Lopez NC et al found that fasudil decreases the function of the RhoA/ROCK pathway, reducing the pulmonary arterial pressure and resistance in chronically hypoxic highland neonatal lambs [34]. Here, we found that RhoA/ROCK pathway was significantly activated in placenta and umbilical vessel of PE mice. And inhibition of RhoA/ROCK pathway with fasudil can reduce the high blood pressure and urine protein levels as well as the concentration of s-Flt in peripheral and umbilical blood in a dose-dependent manner, thus resulting in prevention of the development of PE. However, more studies are needed to evaluate the long term effects of Fasudil treatment on maternal and newborn.

Summary, we here demonstrate that inhibition of ROCK with fasudil can protect vascular endothelial cells from H/R-induced apoptosis and prevent the development of PE. Thus, fasudil would become a potential strategy for PE therapy.

## MATERIALS AND METHODS

### Cell culture and treatment

Human Umbilical Vein Endothelial cells (HUVEC) were obtained from ATCC (American Type Culture Collection, Manassas, VA). The cells were cultured in DMEM supplemented with fetal bovine serum to a final concentration of 10% at 37 °C in a humidified incubator containing 5 % CO2. The cells within 5 passages were used for experiments.

To knock down the level of ROCK II, we transfected ROCK II small interfering RNA (siRNA) into HUVEC cells (RiboBio Co., Ltd, Guangzhou, China) by using Lipofectamine 3000 (Life technologies, Grand Island, NY) in accordance with the manufacture’s instruction. After 48 h transfection, the cells were used for further analysis.

### Hypoxia/reoxygenation model

The hypoxia/reoxygenation model in HUVEC cells was established as we previously described [8]. For hypoxia, the cells were cultured in glucose and serum free media in normal incubator for 30 minutes, the cells then were exposed to hypoxia (1% O_2_) in an AW200SG hypoxic workstation (ELECTROTEK, UK) using a continuous flow of a humidified mixture of 1% O_2_, 5% CO_2_, and 94% N_2_ at 37°C for 4 h. For reoxygenation, after hypoxia, the cells were returned to culture in normal incubator with 20% O_2_, 5% CO_2_, and 75% N_2_ at 37°C for 18 h.

### Determination of IC_50_ of fasudil in HUVEC cells

To determine the IC_50_ of Fasudil, the cells were treated with different concentrations of Fasudil for 8 h, at which time cell proliferation rate were assessed by using MTT. Increasing Fasudil concentrations were designed as 2, 5, 10, 20, 50, 100 and 200 μmol.

### Flow cytometric analysis of apoptosis

Cells from each group were trypsinizated and washed with cold PBS, and then stained by Annexin V-FITC/PI Apoptosis Detection Kit (BD Biosciences) according to the manufacturer’s instructions. The cell apoptosis was analyzed by flow cytometry (Beckman Coulter, USA). The experiments were independently performed in triplicate.

### Preeclampsia-like model preparation

The female C57/BL6N mice were obtained from Hunan SJA Co. (Changsha, China). Animals were housed in a temperature-controlled room (23°C) with a 12:12 light: dark cycle. The experimental animals in this study were approved by the Institutional Animal Care and Research Advisory Committee of Nanjing Medical University. The preeclampsia model was prepared as we previously described. sFlt-1 (Recombinant mouse VEGF R1/Flt-1 Fc Chimera, Cat. No. 7756-FL-050, R&D Systems) was infused at a rate of 3.7 μg/kg/day for 6 days (in sterile saline) beginning on day 7 of gestation via miniosmotic pump (model 2001; Alzet Scientific Corporation, Palto Alto, CA) into pregnant rats. Normal pregnant/control groups (n=8) were fitted with a vehicle filled mini-osmotic pump. The preeclampsia mice were randomly divided into three groups each for 8 animals: PE+ saline, PE+ FSD 10, PE+ FSD 50 and PE+ROCK siRNA. Lentivirus ROCK II siRNA (2 × 10^9^ titer units (TU)/ml, GeneChem Corporation, Shanghai, China) were used to knock down the expression of ROCK II in PE mice. FSD solution (10 or 50 mg/kg/day for 6 days starting on day 13 of gestation, n=8) and lentiviral suspension (100 μl/day for 6 days starting on day 13 of gestation, n=8) were injected into tail veins. The preeclampsia mice treated with saline (n=8) were used as control.

### Blood pressure measurement and tissues collection

Non-invasive tail-cuff Systolic BP measurements were carried out in conscious mice using the non-invasive automated sphygmomanometer (BP-98A, Softron, Beijing, China). The mice were placed in a 38 degree incubator and warmed for 15 minutes to stabilize the mood and to expand the local vessels sufficiently, and then the pressurized sensor was placed in the tail. Mice blood pressure was measured after a few seconds of sobriety and tranquility. Each mouse was continuously measured 5 times and the average was taken as the final result. Systolic BP measurements were performed at the same time to ensure that blood pressure is relatively stable.

The proteinuria and sFlt were measured on day 19. On day 19 of pregnancy, the animals were euthanized under the anesthesia by overdose chloral hydrate. The peripheral and cord blood were collected for further analysis. And placentas and umbilical vessel were collected for western blot and/or IHC analysis.

### Reagents

Primary antibodies: rabbit monoclonal anti-RhoA (cat no. ab187027), rabbit monoclonal anti-ROCK II (cat no. ab71598), rabbit monoclonal anti-ROCK II (phospho Y722) (cat no. ab182648), rabbit monoclonal anti-eNOS (cat no. ab66127), rabbit monoclonal anti-eNOS (phospho S1177) (cat no. ab75639) from Abcam (Cambridge, UK); rabbit polyclonal anti-RhoA (Ser188) (cat no. bs-5330R), rabbit polyclonal anti-MLC (cat no. bs-7115R), rabbit polyclonal anti-phosphorylated MLC (cat no. bs-4060R) and rabbit polyclonal anti-VEGF (cat no. bs-1665R) from Bioss (Beijing, China); mouse monoclonal anti-GAPDH (cat no. SC-365062) were from Santa Cruze (Dallas, Texas, USA). Horseradish peroxidase-conjugated secondary goat anti-mouse and goat anti-rabbit antibodies were from Boster (Wuhan, China). HRP-polymer anti-mouse IHC kit and HRP-polymer anti-rabbit IHC kit were from Maixin (Fuzhou, China). ELISA Kit for soluble fms-like tyrosine kinase-1 (sFlt-1) was from Cloud-Clone Corp. (cat no. SEB818Mu, Houston, TX, USA). Ace (Mouse) ELISA Kit for Angiotensin converting enzyme was from Abnova (cat no. KA3365, Taipei City, Taiwan).

### Immunohistochemical (IHC) staining

The placenta tissues were embedded in paraffin and cut into 4 μm slides. The slides were routinely deparaffinized and hydrated. After inactivating endogenous peroxidase in 3% hydrogen peroxide, slides were then retrieved in citric acid buffer (pH6.0) by microwave for 15 min. Slices were blocked in normal goat serum for 30 min at room temperature, and incubated with primary antibody overnight at 4°C. The slides were then washed with TBST and incubated with appropriate secondary antibody for 2 h at 37°C. The sections were then washed with TBST and stained by using DAB Detection Kit (Solarbio, Beijing, China). Finally, the sections were counterstained with hematoxylin. The slices were captured and analyzed using Motic Images Advanced 3.2 software (Motic, XiaMen, China). The average optical density analysis was conducted by a researcher blind to treatment conditions. Data was acquired from eight sections/animal and the data were averaged to produce the relative expression of proteins.

### Western blot analysis

RIPA lysis buffer was used to extract protein from indicated cells. BCA Protein Assay Kit (Thermo Scientific, USA) was used to measure the protein concentration. Total 60 μg of protein were separated on 10% SDS-PAGE gels and blotted onto nitrocellulose membranes. The membranes were blocked for 2 hours with 5% non fat dry milk diluted with tris buffered saline (TBS) and incubated with primary antibodies overnight at 4°C. The membranes were washed with TBST, and then incubated with appropriate horseradish peroxidase-conjugated secondary antibody for 1 h at 37°C. Enhanced chemiluminescence reagent (Merck Millipore, Germany) was used to detect the signal on the membrane. Western blot bands were quantified by the mean gray value using NIH Image J 7.0. The expression of genes was analyzed by normalizing to the expression of the internal control (GAPDH).

### ELISA

ELISA kits were used to detect the levels of ACE and s-Flt in the serum according to manufacturer’s instructions.

### Statistical analysis

Statistical analyses were performed using Graphpad Prism 5 software (Graphpad Software, Inc., La Jolla, CA, USA) and the data are presented as the mean ± standard deviation. An unpaired two tailed Student’s test or one way analysis of variance (ANOVA) with Bonferroni *t* post-test was used to analyze the data depending on conditions. P<0.05 was considered to indicate a statistically significant difference.
